# Green Synthesis of Silver Nanoparticles by Low-Energy Wet Bead Milling of Metal Spheres

**DOI:** 10.3390/ma13010063

**Published:** 2019-12-21

**Authors:** Andrea Pietro Reverberi, Marco Vocciante, Marco Salerno, Maurizio Ferretti, Bruno Fabiano

**Affiliations:** 1 DCCI, Department of Chemistry and Industrial Chemistry, Università degli Studi di Genova, Via Dodecaneso 31, 16146 Genova, Italy; marco.vocciante@gmail.com (M.V.); ferretti@chimica.unige.it (M.F.); 2 Nanophysics Department, Istituto Italiano di Tecnologia, via Morego 30, 16163 Genova, Italy; marco.salerno@iit.it; 3 DICCA, Department of Civil, Chemical and Environmental Engineering, Polytechnic School, Università degli Studi di Genova, Via Opera Pia 15, 16145 Genova, Italy; brown@unige.it

**Keywords:** nanoparticle synthesis, bead milling, top-down method, eco-friendly process, green nanotechnology

## Abstract

A low-energy, magnetically-driven milling technique for the synthesis of silver nanoparticles is proposed, where the grinding medium and the metal precursor consisting of silver spheres have the same shape and size, belonging to a millimetric scale. The process is carried out at room temperature in aqueous solvent, where different types of capping agents have been dissolved to damp particle agglomeration. The particle diameters, determined by dynamic light scattering and transmission electron microscopy, have been compared with those typical of conventional wet-chemical bottom-up synthesis processes. The use of milling spheres and metal precursor of the same initial shape and size allows to overcome some drawbacks and limitations distinctive of conventional bead-milling equipment, generally requiring complex operations of separation and recovery of milling media. The milling bead/nanoparticle diameter ratio obtained by this approach is higher than that typical of most previous wet bead milling techniques. The method described here represents a simple, one-pot, cost-effective, and eco-friendly process for the synthesis of metal nanoparticles starting from a bulky solid.

## 1. Introduction

**Figure 1:**
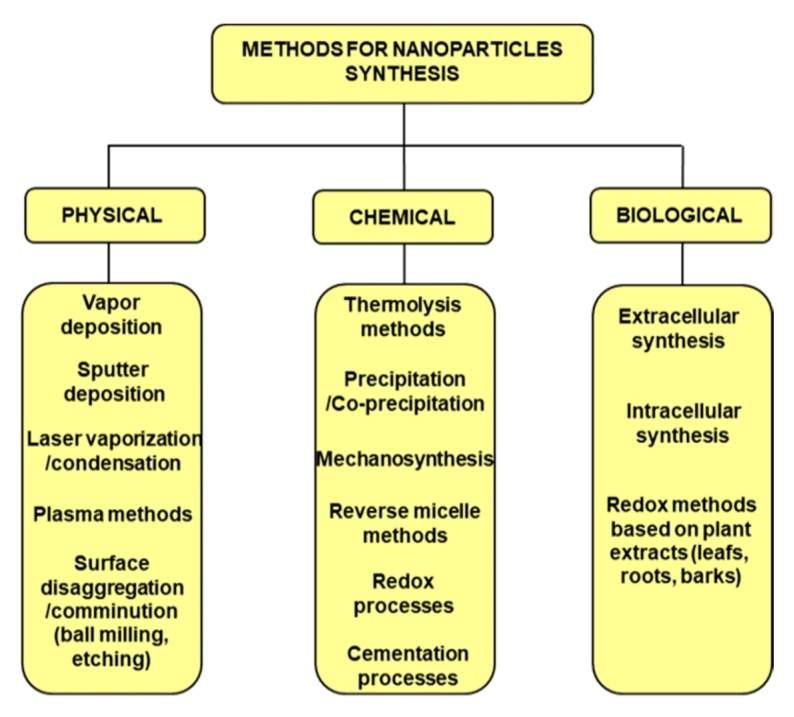
Scheme of the main methods adopted for nanoparticles (NPs) synthesis.

In recent years, the methods for the synthesis of nanoparticles (NPs) have received progressively growing attention and many techniques have been refined according to the physico-chemical properties of the specific NPs, as indicated in the scheme reported in Figure 1. Many manufacturing processes have been improved and implemented on large-scale facilities, as in the production of nanosized alumina, zirconia, and other inorganic compounds like ceramic materials [1]. In other cases, the processes are still limited to a smaller scale, typical of pilot plants or even a laboratory environment [2], owing to constraints related to safety [3], lack of process control, and cost of unit operations.

Very often, chemical methods are a first-rank choice in several synthesis techniques concerning the production of nanosorbents for environmental remediation [4], the preparation of nitrogen-doped graphene (NG) as a metal-free carbon-based catalyst to degrade recalcitrant organics [5], the synthesis of nanoceramics for application in dentistry [6], the fabrication of nanosized sensors to ensure safety conditions in oxidation processes [7], and the production of zerovalent NPs by redox processes [8]. The latter are generally carried out in liquid phases [9], where a precursor containing the ion of the element forming the nanoparticles is dissolved and mixed with a proper electron-donor carrying out the reduction process. An exhaustive discussion about redox methods for the production of NPs for weak and strong electropositive cations can be found in the review papers of Daruich De Sousa et al. [10] and Stefaniuk et al. [11], respectively, and we refer the reader to the references quoted there. The main drawback related to the aforementioned bottom-up redox methods depends on the use of organic or inorganic reductants with toxic effects on humans and the environment. The paper of Rodrigues et al. [12] is a very exhaustive survey concerning a wide choice of electron-donors used in metal nanocrystal synthesis. Among them, hydrazine and its compounds, despite its excellent properties as a reducing agent, joined with a relatively low cost are subject to safety restrictions for their cytotoxic, mutagen, and carcinogenic activity, both on microorganisms and vertebrates [13]. For these reasons, a strategy based on reagent substitution protocols is becoming a paradigm in eco-friendly NPs synthesis [14], as proven by the recent development of green methods based on reagents and capping agents of biological origin [15] and by the overall trend towards the neologistic “green nanotechnology” [16]. As an example, this aspect finds important applications in heat transfer technology [17], where nanofluids as heat carriers need to combine a low toxicity with an efficient stabilization against agglomeration for a wide range of temperature and fluid-dynamics conditions [18]. Nowadays, many reagents derived from plant extracts, pigments, yeast, and enzymes have been used as reductants for the synthesis of zerovalent NPs [19], with more positive results in cases of precursors containing noble metals like gold, platinum, palladium, silver [20], and other elements of weakly electropositive properties [21]. Top-down chemical methods, though representing a cost-effective alternative strategy for the production of inorganic nanoparticles, are still confined to a narrow field, probably owing to a lack of control on particle diameters, whose broad distribution adversely affects the quality of the final product. In some cases, a combination of top-down etching methods with traditional bottom-up routes led to promising results in surface patterning and functionalization [22].

A further step forward in the minimization of environmental impact of NPs manufacturing is offered by top-down physical methods [23], in which a nanomaterial is formed starting from elements or compounds in macroscopic sizes undergoing treatments without any chemical reaction. Several disaggregation techniques have been proposed [24], whose classification generally depends on the presence/absence of milling media like spheres or beads in a vessel containing the phase subject to comminution [25]. The effect of mechanical impact and friction may be advantageously controlled by the use of proper capping agents damping the simultaneous reaggregation tendency of the as-produced nanoparticles [26]. In some cases, as in cryomilling processes, the presence of such stabilizing agents is avoided in order to obtain a nanophase totally free of surface contaminants [27]. 

The present work belongs to the context of top-down physical methods, in that it deals with the formation of nanosized particles of zerovalent Ag through disaggregation of silver spheres, whose surface is subject to impact and abrasion carried out by ceramic balls of yttrium-stabilized zirconia dispersed in aqueous solvent where capping agents have been previously dissolved. The choice of the aforementioned grinding medium is motivated by its high density, negligible surface porosity, and peculiarity of having an increasing tenacity with a growing number of collision events. The presence of a small concentration of Y_2_O_3_ has a limited effect on the cost of such ceramic balls.

Essentially, the main purpose of this work is to create a dispersion of Ag NPs in a one-pot process, using both a metal precursor (Ag) and a milling medium with diameters at a millimetric scale. This strategy may represent an alternative to conventional wet bead millers, which are generally designed to break NPs agglomerates as a source material, rather than producing NPs starting from a bulky phase, as in the case proposed here. The paper is divided as follows. In Section 2, the process of synthesis is described and some details concerning the experimental set-up are given. In Section 3, the results are presented and related to the previous outcomes in literature concerning wet disaggregation techniques. In Section 4, the conclusions are drawn and the direction for future work is traced.

## 2. Materials and Methods

### 2.1. Composition of the Vessel Content

Milli-Q water was used as solvent in all experimental samples.

Silver spheres (Ag, 99.9%, American Elements, Los Angeles, CA, USA) were adopted as metal precursor undergoing disaggregation. Yttria-stabilized zirconia spheres (ZrO_2_ 95%, Y_2_O_3_ 5%, MSE Supplies, Tucson, AZ, USA) were used as supplied.

The list of capping agent adopted here comprises urea (UR, CO(NH_2_)_2_, 99%, La Farmochimica, Genova, Italy); tetrabutylammonium bromide (TBAB, (C_4_H_9_)_4_NBr, 99%, Merck, Milano, Italy); sodium dodecyl sulphate (SDS, CH_3_(CH_2_)_11_OSO_3_Na, 99%, Sigma-Aldrich, Milano, Italy); *N*,*N*-dimethyldodecylamine N-oxide (DDAO, CH_3_(CH_2_)_11_NO(CH_3_)_2_, 30% in water, Sigma-Aldrich, Milano, Italy); and three different forms of polyvinyl pyrrolydone (PVP, (C_6_H_9_NO)_n_, 99%, Sigma-Aldrich, Milano, Italy), with 10 kDa, 40 kDa, and 750 kDa as average molecular weights.

In the following, the experimental samples are labelled with letters *a*–*g* according to the type of capping agent adopted, as reported in Table 1. PVP was chosen in three different compositions to ascertain the effect of its different molecular weights on the final product. The concentration of capping agents for samples *a*,*b*,*c* was fixed at 0.2 g in 3 cm^3^ of liquid hold-up, in order to ensure the same number of monomers for each sample. For samples *d*,*e*,*f*, 0.1 g in 3 cm^3^ of liquid hold-up was set. For sample *g*, the concentration was 0.3 cm^3^ in 3 cm^3^ of water.

### 2.2. The Experimental Set-up

**Figure 2:**
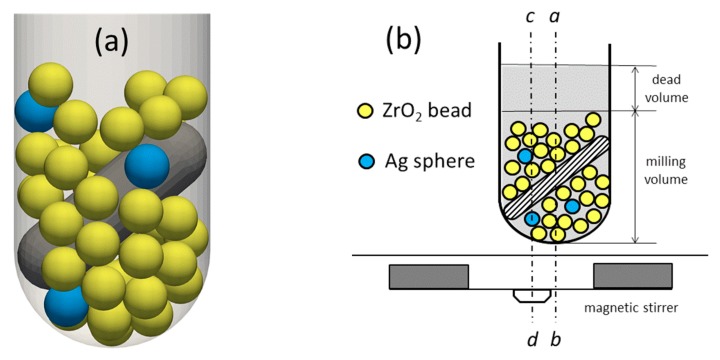
(**a**) Scheme of the miller assembly with the grey stirring bar in skew position (adapted from the work of [30]); (**b**) scheme indicating the position of the vessel axis *ab* with respect to the axis *cd* of the magnetic stirring disc.

The home-made stirring/milling equipment comprises a round bottom cylindrical vessel of 13.5 cm^3^ volume, where a polytetrafluoro ethylene (PTFE)-coated magnetic stirring bar, whose length is greater than the inner diameter of the vessel, is forced to rotate in a skew position with respect to the axis *ab* of the vessel by means of a magnetic stirrer, located at the bottom of the container schematically represented in Figure 2a. In each experimental sample, besides the stirring bar (15 mm length; 4.5 mm diameter), the vessel contains the following:

- 3 cm^3^ of solvent, where the corresponding mass of capping agent was previously dissolved;
- 40 ZrO_2_/Y_2_O_3_ ceramic spheres of 3 mm diameter, forming a bed in static conditions whose height must allow the rotation of the stirrer without jams;
- three silver spheres of 3 mm diameter.

There is not a common opinion about the maximum allowed diameter of grinding spheres in a bead milling process, but a diameter of 3 mm adopted in the present study can be considered as an upper threshold value [28].

Two cylindrical magnets, positioned on a disk below the flat surface of the stirrer in Figure 2b, turn around the axis *cd* with adjustable speed, which was maintained at 600 rpm in all experiments.

A considerable lack of milling efficiency was observed when the axes *ab* and *cd*, drawn as dashed lines in Figure 2b, overlap one another. This phenomenon can be ascribed to a drop of frequency of collisional events between spheres, irrespective of their composition. For this reason, in the present experimental campaign, the axis *ab* was shifted by approximately 5 mm in parallel to the axis *cd*.

The vessel is gas-tight to avoid the evaporation of the solvent, but the process is carried out in the presence of air. On the opposite, previous studies showed that the use of inert gases is necessary when zerovalent NPs of non-noble metals are produced, owing to their high tendency for oxidation [29]. Unlike many high-energy milling processes, no noticeable temperature rise was detected here during the milling process, and hence no cooling equipment was necessary. After 5 h of milling time for all experimental samples, the liquid hold-up is allowed to stand for 12 h and it is further centrifuged at 8000 rpm for 15 min. The supernatant is then separated and analyzed with the instruments described in the next section.

### 2.3. Characterization Techniques

The average hydrodynamic size of the fabricated Ag NPs was obtained by carrying out dynamic light scattering (DLS) in the surfactant-modified aqueous media of the respective samples. We used a Zetasizer Nano instrument (Malvern, UK), equipped with a red light source. The cuvettes were disposable polystyrene (VWR, Leuven, Belgium), 2.5 mL in size, filled in with approximately 1.2 mL suspension. The acquisition was run in non-invasive backscattering mode (173°), typically based on three measurements of seven runs each (run duration 10 s, no delay in between), started after 120 s sample equilibration time at 25 °C. We used the general purpose model of time autocorrelation in the Zetasizer V.7.11 software (no protein analysis). For the optical properties of the Ag NPs, we used a refractive index of 0.2 and an absorption coefficient of 0.6. Given the little amount of surfactants, the dispersant was always modeled as pure water, with a refractive index of 1.33 and viscosity of 0.8872 cP. After running the measurement, the raw data of size distribution by scattered light intensity are automatically converted—via the built-in model—to size distribution by volume, and finally to size distribution by number (%), which is what is reported in the figures. The absolute count rate was always in the order of 200 kcps.

High resolution imaging of the Ag NPs was carried out by transmission electron microscopy (TEM) on an instrument JEM-1011 (Jeol, Japan), operating at 100 kV. The solutions of NPs were spread on copper grids coated with carbon films. Compositional analysis of the NPs was performed by energy-dispersive X-ray spectroscopy (EDS) carried out in a scanning electron microscope (SEM) JSM-6490LA (Jeol, Japan), working at 15 kV. The solutions of NPs were cast on an aluminium stub and coated with 30 nm carbon.

## 3. Results and Discussion

**Figure 3:**
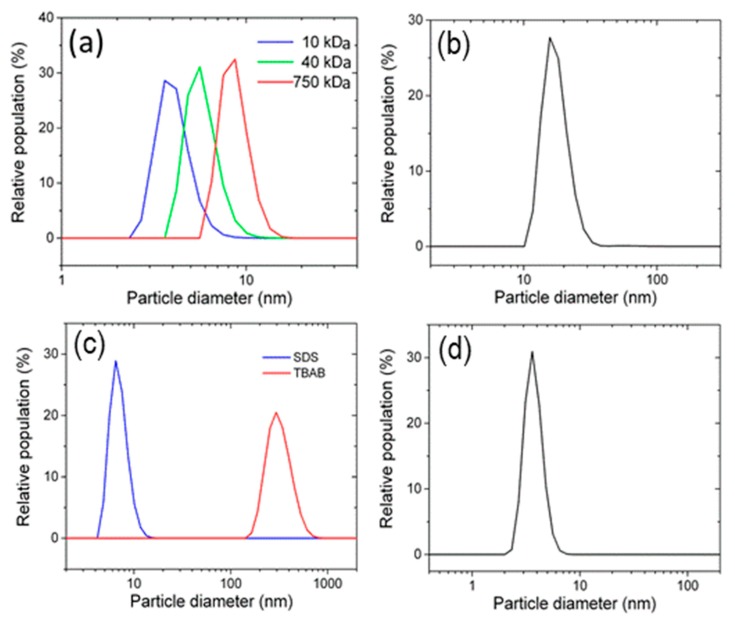
Dynamic light scattering (DLS) measurements of the mean nanoparticle (NP) size resulting from the use of different surfactants. (**a**) Polyvinyl pyrrolydone (PVP) of various molecular weight (MW); (**b**) urea (UR); (**c**) sodium dodecyl sulphate (SDS) and tetrabutylammonium bromide (TBAB); (**d**) *N*,*N*-dimethyldodecylamine N-oxide (DDAO).

In Figure 3, the probability distribution of particle diameters is reported for different groups of capping agents. The curves in the upper left plot (a) refer to three different molecular weights of PVP, whose properties as stabilizer, grafting agent [31], and size-orienting component have been amply recognized and investigated in the literature, both for Ag and for different zerovalent NPs made of non-transition elements [32]. It can be seen that the smallest average particles diameters for non-ionic dispersants are obtained for PVP 10k, namely in the case of shorter polymer chains. Intriguingly, this trend is the opposite of what has been found in the literature concerning the influence of PVP molecular weight on the dimension of Ag NPs synthesized by wet chemical [33] and radiochemical methods [34]. The rationale behind the trend observed here could be found in the mechanism of formation and further stabilization of the NPs in a physical top-down disaggregation process, whose steps differ from those typical of chemically driven bottom-up synthesis methods. In this specific case of non-ionic stabilization, it is supposed that PVP chains of lower molecular weight could more efficiently cover the surface of silver spheres, thus promoting a better stabilization of the clusters detached by shear stress and collision from the metal surface itself. This aspect will be reconsidered at the end of this section.

In Figure 3b, analogously, the distribution of Ag NPs diameters is reported using urea as capping agent. In this case, Ag NPs with average diameters of 16 nm were obtained, proving that urea has lower efficiency with respect to PVP of any molecular weight, when lower NPs’ diameters are desired.

In Figure 3c, the effect of ionic capping agents is described, showing a markedly higher efficiency of the anionic stabilizer (SDS), giving 6.5 nm as the average diameter, a value considerably smaller than that obtained with the cationic one (TBAB). In the latter case, only a microsized solid phase was produced with an average diameter of 300 nm, namely larger than those obtained in all other experimental runs.

Figure 3d refers to a Zwitterionic capping agent, a proven choice for the synthesis of Ag nanowires [35] and, in more recent times, for antibacterial surface functionalization by co-deposition of Ag NPs with Zwitterionic polymers [36]. In the present bead milling process, DDAO showed a high efficiency as stabilizer, leading to a dispersion of Ag NPs with an average diameter of 3.5 nm.

Despite the great amount of literature concerning Ag NPs bottom-up synthesis by chemical reduction, a definitive conclusion about the superiority of cationic or anionic stabilizers in that context cannot be easily drawn, as the polarity of the capping agent may even interfere with the thermodynamic and kinetics of the process leading from Ag^+^ to Ag^(0)^ [37]. Furthermore, as far as Ag NPs stabilization in chemical synthesis is concerned, the following was observed:

- cationic stabilizers have a well-defined shape-orienting effect [38], generally greater than that typical of SDS [37];
- electrostatic stabilization by ionic surfactants proved to be more efficient than steric stabilization by non-ionic surfactants [39].

The latter consideration does not seem to hold for the stabilization of Ag NPs produced by a purely physical synthesis method as the one discussed here. In fact, the curves reported in snapshots (a) and (b) of Figure 3, concerning non-ionic capping agents like PVP and urea, lead to average mean diameters being comparable, and in some cases, better than those typical of ionic stabilizers. The lowest average particle diameter obtained with PVP 10k is consistent with the high efficiency of the ketonic group as an electron-pair donor to zerovalent Ag. The effects of different capping agents on Ag NPs average diameters estimated by DLS measurements are reported in Table 2.

**Figure 4:**
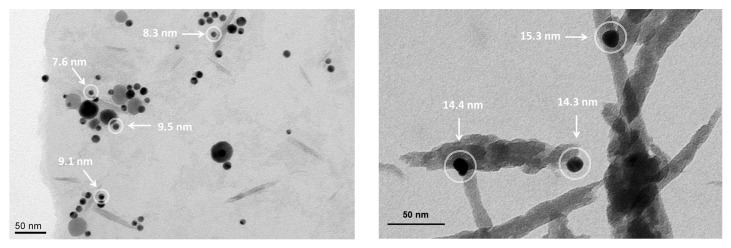
Transmission electron microscopy (TEM) images of samples obtained using PVP 10k and UR as capping agents in the left and right panels, respectively.

In Figure 4, TEM images of Ag NPs obtained from samples *a* and *d* of Table 1 are reported. In left panel, the NPs diameters are statistically higher than those measured by DLS counting for PVP 10k, but many highlighted NPs have diameters <10 nm, in agreement with the corresponding distribution curve of Figure 3a. In right panel, the three NPs visualized in white circles have diameters >10 nm, and these values are consistent with the distribution curve of Figure 3b pertaining to UR as capping agent.

**Figure 5:**
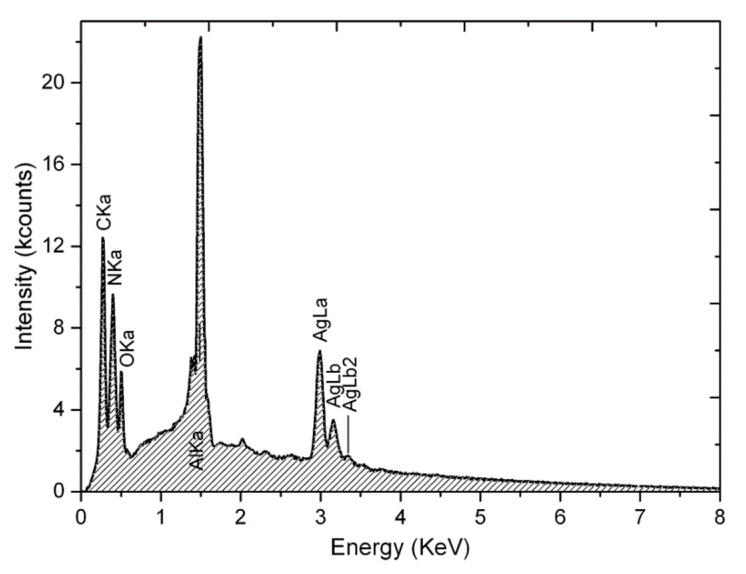
Energy-dispersive X-ray spectroscopy (EDS) spectrum acquired on an aggregate of Ag NPs drop cast from a solution containing UR (scanning electron microscope (SEM) image not shown). The presence of metallic Ag is clearly assessed.

Typical qualitative results of EDS compositional analysis are reported in Figure 5, for the case of Ag NPs drop cast from a solution containing UR. From the X-ray background of Bremsstrahlung, Ag peaks clearly emerge, together with C, from both UR and, above all, the SEM drain layer coating and N from UR. The highest peak is from the Al substrate of the stub, given the deep penetration of the primary electrons in the beam, while residual O also appears, which is owing to both UR and the oxidized Al stub surface, but is clearly below levels consistent with the presence of Ag oxide.

What has been stated up to now in the present section can be summarized in the following points:

- Except for the case of cationic stabilizer (TBAB), Ag NPs of small average diameters (<16 nm) were obtained in essentially all cases proposed here according to DLS data, with small relative differences between samples, irrespective of the chemical structure and/or functional groups of the corresponding capping agent;
- As a consequence of the above point, this case of top-down disaggregation process describes a new scenario with respect to conventional wet-chemical synthesis, where capping agents differing in chemical composition show remarkable variations in efficiency.

A possible qualitative explanation of such intriguing aspects could be given by taking into account the sequence of dynamic events causing a basic difference between a bottom-up chemical and a top-down physical synthesis process, like that typical of the present study.

In the former, metal ions generate zerovalent atoms, whose concentration grows in time until it reaches a threshold value, triggering the process of aggregation and finally the Ostwald ripening, namely the coarsening mechanism by which larger particles grow by incorporating smaller ones [40]. Moreover, it was observed that surfactants or capping agents are active since the first step of the global process, as they tend to form bonds both with ionic species as well as with nuclei during their initial process of aggregation. [41].

**Figure 6:**
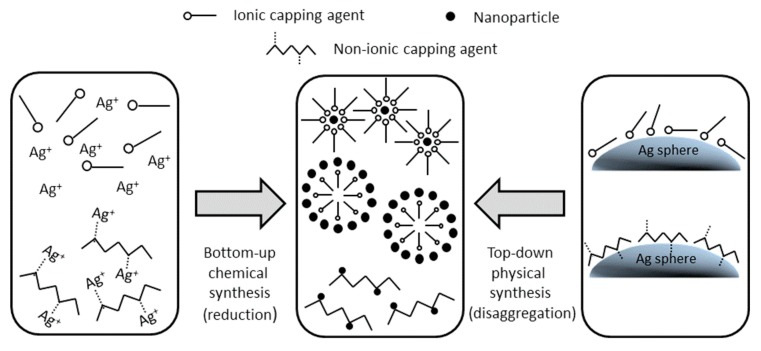
Scheme of different patterns for the synthesis of metal NPs in the case of wet-chemical bottom-up processes and in the case of top-down physical disaggregation by wet bead milling (the present study).

In the latter, an ionic species at the start is present at a negligible concentration related to the thermodynamic equilibria of metal/ion in a solvent, while micro- and nanosized surface elements are progressively detached from the metallic surface by mechanical friction; abrasion; or, more rarely in a low-energy milling, by shock. For these reasons, the mechanisms and the kinetic steps of surface stabilization by capping agents in bottom-up chemical synthesis are deeply different from those occurring in top-down physical synthesis processes, as schematically indicated in Figure 6. The modelling of such different scenarios has been extensively developed in chemical synthesis [42], while it has received very limited attention in bead milling processes until now.

As anticipated in the Introduction, milling processes for NPs manufacturing are currently in the hotspot for their sustainability and versatility in drug preparation [43] and in mechanochemical synthesis [44]. Ogi et al. [24] authored an interesting review paper containing a systematic classification of machining processes for the production of NPs dispersions by milling methods. Among them, wet bead-milling continuous processes are extensively analyzed and many details concerning different technical solutions are presented, essentially differing from one another about the realization of the rotor pin and of the centrifugal separator between beads and NPs. In particular, the authors pointed out the difference between media-less and media-assisted methods in top-down disaggregation. Among the latter ones, it is worth reminding that low-energy bead-milling/bead-stirring apparatuses are generally used to decrease the diameter of particles already belonging to a micron or even submicron range. This amounts to saying that such processes are essentially designed to disaggregate NP agglomerates [45], thereby releasing primary particles, and this explains why bead millers are fed with liquids containing suspended particles, even forming nanodispersions at the start [46], as well known in pharmaceutical science [47]. A crucial aspect in these processes is the establishment of an optimal matching between the size of the solid phase feeding the miller and the diameter of the beads carrying out the disaggregation, as a relatively large inter-bead space at a close-packing configuration may lead to a dramatic loss of milling efficiency [48]. In a previous work of ours [49], a nanophase made of primary particles was produced in a two-pot process, where agglomerated crystals of zerovalent bismuth obtained by chemical cementation underwent a wet bead-milling to reach a final state of stable nanometric dispersion.

On the opposite, studies proposing a one-pot bead milling process where NPs are produced directly starting from a bulky solid like metal spheres of millimetric dimension are quite uncommon in the literature. The present work is an example of such an approach, with a magnetically-driven motion of both precursor and milling spheres. In a somewhat different context, Romero et al. [50] proposed a miniaturized and magnetically driven wet bead miller to comminute an initially microcrystalline phase in a batch process, with beads’ diameters spanning in the range of 0.05–0.6 mm. Their results essentially confirmed the well-known rule of thumb, stating that the minimum achievable final diameter of particles may reach 1/1000 of the bead diameter in wet bead milling equipment.

In the present non-conventional bead milling technique, the aforementioned rule seems to be violated by more than two orders of magnitude. In fact, in all cases but one of Ag disaggregation carried out in aqueous solvent, whatever the capping agent, the nanoparticle diameters are smaller than 30 nm, a value corresponding to a 1/10^5^ ratio between Ag NP and bead diameters. A possible explanation could be given taking into account the following points, which highlight the main differences between the present disaggregation method and the standard bead milling processes:

- The aforementioned rule of “1/1000” has proven its validity in the presence of an initial solid phase made of agglomerated crystals or particles having microsized dimensions. The present case, instead, concerns a compact homogeneous solid phase of a bulky metal.
- Unlike the scheme of Romero et al. [50], the present disaggregation process occurs primarily at the bottom of the vessel, as depicted in Figure 2b, owing to the weight of both beads and metal spheres. In this volume, the metal–bead friction is particularly active, leading to a metal surface disaggregation with release of micro- and nanoparticles. Here, the stirring bar turns along the generatrix of a cone, creating zones of null collision beside zones with a high probability of collisions among spheres, thus enhancing the velocity gradients and ultimately the shear stresses on Ag spheres.
- The dead volume at the top of the vessel is now beneficial, acting like a sort of escaping zone for the nanoparticles, subtracting them from the milling volume and thus reducing their probability of sintering and reagglomeration. This new aspect might explain why, in the present study, NPs of considerably small diameters were obtained in almost all cases, irrespective of the capping agent used.

Mc Mahon et al. [51] addressed the problem of whether a capping agent directly acts on the surface of a bulk solid subject to disaggregation. They synthesized Al, Cu, and Fe NPs in a planetary miller using different solvents and surfactants. The highest NPs/microparticles mass ratio was obtained using solvent forming bonds or having affinities with the metal surface. More intriguingly, oleic acid acted as one of the most efficient capping agents in promoting the formation of metal NPs, despite its lubricating properties. It was conjectured that a basic role in such a process is played by the adsorption of solvent or surfactant on the metal surface, whose free energy is lowered, thus enhancing its chemisorption-induced decohesion and finally its disaggregation [52]. Admittedly, in agreement with the latter consideration, analogous mechanism can be invoked in this case of low-energy disaggregation, to explain an unexpected synthesis of metal NPs starting from a bulky solid.

## 4. Conclusions

A bead-milling process for the synthesis of Ag NPs using Ag spheres as source material was proposed. In terms of practical relevance, such a method may represent an alternative to chemical routes in the pharmaceutical industry whenever the preparation of antiseptic/antibacterial solutions is required in emergency situations, thanks to the small scale and portability of the experimental setup described here.

From a methodological point of view, the key aspects of the present work can be summarized as follows:

- Ag NPs were obtained by a simple, non-conventional, and cost-effective bead milling apparatus based on a low-energy disaggregation process. The miller does not need a moving shaft of complex shape with gas-tight fittings, neither built-in centrifugal separators for milling bead recovery, nor additional temperature control devices, with a considerable simplification of the miller setup, compared with the usually tricky bead-milling solutions.
- The requirements of eco-compatibility are met in this simple top-down physical process where a precursor at its elemental state is used, as no toxic or noxious chemical reactant is needed. The apparatus is scalable, within the limits imposed by the efficiency of a magnetic stirrer in larger plants.
- Excluding the case concerning a cationic stabilizer, Ag NPs with average diameters <16 nm were obtained with all the other capping agents, proving the global reliability of the process in terms of quality of the final product.
- Tuning the shape of NPs produced by the present set-up is not considered so far. It can be conjectured that temperature may have an important role on the surface adsorption of each capping agent on the bulk metal surface, thereby conditioning the shape of the as-made NPs.
- To the best of our knowledge, this is one of the very few wet bead-milling processes where the diameter of the milled phase gets nanosized by means of the milling media and precursor both belonging to a millimetric scale.

A future development of such process will be aimed at synthesizing zerovalent NPs of non-noble metals for toxic waste decontamination.

## Figures and Tables

**Table 1:** List of experimental samples and their composition. PVP, polyvinyl pyrrolydone; UR, urea; TBAB, tetrabutylammonium bromide; SDS, sodium dodecyl sulphate; DDAO, *N*,*N*-dimethyldodecylamine N-oxide.

Sample Label	Capping Agent	Type of Capping Agent
*a*	PVP 10k	Non ionic
*b*	PVP 40k	Non ionic
*c*	PVP 750k	Non ionic
*d*	UR	Non ionic
*e*	TBAB	Cationic
*f*	SDS	Anionic
*g*	DDAO	Zwitterionic

**Table 2:** Average Ag nanoparticles’ (NPs’) diameters obtained with different capping agents.

Capping Agent	Ag-NPs’ Average Diameter (nm)
PVP 10k	3.6
PVP 40k	5.6
PVP 750k	8.7
UR	16
TBAB	300 (microparticles)
SDS	6.5
DDAO	3.5

## Abbreviations

The following abbreviations are used in this manuscript:

cP	centiPoise
cps	counts per second
Da	Dalton
DDAO	*N*,*N*-dimethyldodecylamine N-oxide
DLS	dynamic light scattering
EDS	energy dispersive X-ray spectroscopy
MW	molecular weight
NG	nitrogen-doped graphene
NPs	nanoparticles
PVP 10k	polyvinyl pyrrolydone with 10 kDa molecular weight
PVP 40k	polyvinyl pyrrolydone with 40 kDa molecular weight
PVP 750k	polyvinyl pyrrolydone with 750 kDa molecular weight
PTFE	polytetrafluoro ethylene
SDS	sodium dodecyl sulphate
SEM	scanning electron microscopy
TBAB	tetrabutylammonium bromide
TEM	transmission electron microscopy
UR	urea
